# Measurement of the $t\bar{t}$ production cross section in the tau + jets channel using the ATLAS detector

**DOI:** 10.1140/epjc/s10052-013-2328-7

**Published:** 2013-03-02

**Authors:** The ATLAS Collaboration, G. Aad, T. Abajyan, B. Abbott, J. Abdallah, S. Abdel Khalek, A. A. Abdelalim, O. Abdinov, R. Aben, B. Abi, M. Abolins, O. S. AbouZeid, H. Abramowicz, H. Abreu, B. S. Acharya, L. Adamczyk, D. L. Adams, T. N. Addy, J. Adelman, S. Adomeit, P. Adragna, T. Adye, S. Aefsky, J. A. Aguilar-Saavedra, M. Agustoni, M. Aharrouche, S. P. Ahlen, F. Ahles, A. Ahmad, M. Ahsan, G. Aielli, T. P. A. Åkesson, G. Akimoto, A. V. Akimov, M. A. Alam, J. Albert, S. Albrand, M. Aleksa, I. N. Aleksandrov, F. Alessandria, C. Alexa, G. Alexander, G. Alexandre, T. Alexopoulos, M. Alhroob, M. Aliev, G. Alimonti, J. Alison, B. M. M. Allbrooke, L. J. Allison, P. P. Allport, S. E. Allwood-Spiers, J. Almond, A. Aloisio, R. Alon, A. Alonso, F. Alonso, A. Altheimer, B. Alvarez Gonzalez, M. G. Alviggi, K. Amako, C. Amelung, V. V. Ammosov, S. P. Amor Dos Santos, A. Amorim, N. Amram, C. Anastopoulos, L. S. Ancu, N. Andari, T. Andeen, C. F. Anders, G. Anders, K. J. Anderson, A. Andreazza, V. Andrei, M-L. Andrieux, X. S. Anduaga, S. Angelidakis, P. Anger, A. Angerami, F. Anghinolfi, A. Anisenkov, N. Anjos, A. Annovi, A. Antonaki, M. Antonelli, A. Antonov, J. Antos, F. Anulli, M. Aoki, S. Aoun, L. Aperio Bella, R. Apolle, G. Arabidze, I. Aracena, Y. Arai, A. T. H. Arce, S. Arfaoui, J-F. Arguin, S. Argyropoulos, E. Arik, M. Arik, A. J. Armbruster, O. Arnaez, V. Arnal, A. Artamonov, G. Artoni, D. Arutinov, S. Asai, S. Ask, B. Åsman, L. Asquith, K. Assamagan, A. Astbury, M. Atkinson, B. Aubert, E. Auge, K. Augsten, M. Aurousseau, G. Avolio, D. Axen, G. Azuelos, Y. Azuma, M. A. Baak, G. Baccaglioni, C. Bacci, A. M. Bach, H. Bachacou, K. Bachas, M. Backes, M. Backhaus, J. Backus Mayes, E. Badescu, P. Bagnaia, S. Bahinipati, Y. Bai, D. C. Bailey, T. Bain, J. T. Baines, O. K. Baker, M. D. Baker, S. Baker, P. Balek, E. Banas, P. Banerjee, Sw. Banerjee, D. Banfi, A. Bangert, V. Bansal, H. S. Bansil, L. Barak, S. P. Baranov, T. Barber, E. L. Barberio, D. Barberis, M. Barbero, D. Y. Bardin, T. Barillari, M. Barisonzi, T. Barklow, N. Barlow, B. M. Barnett, R. M. Barnett, A. Baroncelli, G. Barone, A. J. Barr, F. Barreiro, J. Barreiro Guimarães da Costa, R. Bartoldus, A. E. Barton, V. Bartsch, A. Basye, R. L. Bates, L. Batkova, J. R. Batley, A. Battaglia, M. Battistin, F. Bauer, H. S. Bawa, S. Beale, T. Beau, P. H. Beauchemin, R. Beccherle, P. Bechtle, H. P. Beck, K. Becker, S. Becker, M. Beckingham, K. H. Becks, A. J. Beddall, A. Beddall, S. Bedikian, V. A. Bednyakov, C. P. Bee, L. J. Beemster, M. Begel, S. Behar Harpaz, P. K. Behera, M. Beimforde, C. Belanger-Champagne, P. J. Bell, W. H. Bell, G. Bella, L. Bellagamba, M. Bellomo, A. Belloni, O. Beloborodova, K. Belotskiy, O. Beltramello, O. Benary, D. Benchekroun, K. Bendtz, N. Benekos, Y. Benhammou, E. Benhar Noccioli, J. A. Benitez Garcia, D. P. Benjamin, M. Benoit, J. R. Bensinger, K. Benslama, S. Bentvelsen, D. Berge, E. Bergeaas Kuutmann, N. Berger, F. Berghaus, E. Berglund, J. Beringer, P. Bernat, R. Bernhard, C. Bernius, T. Berry, C. Bertella, A. Bertin, F. Bertolucci, M. I. Besana, G. J. Besjes, N. Besson, S. Bethke, W. Bhimji, R. M. Bianchi, L. Bianchini, M. Bianco, O. Biebel, S. P. Bieniek, K. Bierwagen, J. Biesiada, M. Biglietti, H. Bilokon, M. Bindi, S. Binet, A. Bingul, C. Bini, C. Biscarat, B. Bittner, C. W. Black, K. M. Black, R. E. Blair, J.-B. Blanchard, T. Blazek, I. Bloch, C. Blocker, J. Blocki, A. Blondel, W. Blum, U. Blumenschein, G. J. Bobbink, V. S. Bobrovnikov, S. S. Bocchetta, A. Bocci, C. R. Boddy, M. Boehler, J. Boek, T. T. Boek, N. Boelaert, J. A. Bogaerts, A. Bogdanchikov, A. Bogouch, C. Bohm, J. Bohm, V. Boisvert, T. Bold, V. Boldea, N. M. Bolnet, M. Bomben, M. Bona, M. Boonekamp, S. Bordoni, C. Borer, A. Borisov, G. Borissov, I. Borjanovic, M. Borri, S. Borroni, J. Bortfeldt, V. Bortolotto, K. Bos, D. Boscherini, M. Bosman, H. Boterenbrood, J. Bouchami, J. Boudreau, E. V. Bouhova-Thacker, D. Boumediene, C. Bourdarios, N. Bousson, A. Boveia, J. Boyd, I. R. Boyko, I. Bozovic-Jelisavcic, J. Bracinik, P. Branchini, A. Brandt, G. Brandt, O. Brandt, U. Bratzler, B. Brau, J. E. Brau, H. M. Braun, S. F. Brazzale, B. Brelier, J. Bremer, K. Brendlinger, R. Brenner, S. Bressler, T. M. Bristow, D. Britton, F. M. Brochu, I. Brock, R. Brock, F. Broggi, C. Bromberg, J. Bronner, G. Brooijmans, T. Brooks, W. K. Brooks, G. Brown, P. A. Bruckman de Renstrom, D. Bruncko, R. Bruneliere, S. Brunet, A. Bruni, G. Bruni, M. Bruschi, L. Bryngemark, T. Buanes, Q. Buat, F. Bucci, J. Buchanan, P. Buchholz, R. M. Buckingham, A. G. Buckley, S. I. Buda, I. A. Budagov, B. Budick, V. Büscher, L. Bugge, O. Bulekov, A. C. Bundock, M. Bunse, T. Buran, H. Burckhart, S. Burdin, T. Burgess, S. Burke, E. Busato, P. Bussey, C. P. Buszello, B. Butler, J. M. Butler, C. M. Buttar, J. M. Butterworth, W. Buttinger, M. Byszewski, S. Cabrera Urbán, D. Caforio, O. Cakir, P. Calafiura, G. Calderini, P. Calfayan, R. Calkins, L. P. Caloba, R. Caloi, D. Calvet, S. Calvet, R. Camacho Toro, P. Camarri, D. Cameron, L. M. Caminada, R. Caminal Armadans, S. Campana, M. Campanelli, V. Canale, F. Canelli, A. Canepa, J. Cantero, R. Cantrill, M. D. M. Capeans Garrido, I. Caprini, M. Caprini, D. Capriotti, M. Capua, R. Caputo, R. Cardarelli, T. Carli, G. Carlino, L. Carminati, B. Caron, S. Caron, E. Carquin, G. D. Carrillo-Montoya, A. A. Carter, J. R. Carter, J. Carvalho, D. Casadei, M. P. Casado, M. Cascella, C. Caso, A. M. Castaneda Hernandez, E. Castaneda-Miranda, V. Castillo Gimenez, N. F. Castro, G. Cataldi, P. Catastini, A. Catinaccio, J. R. Catmore, A. Cattai, G. Cattani, S. Caughron, V. Cavaliere, P. Cavalleri, D. Cavalli, M. Cavalli-Sforza, V. Cavasinni, F. Ceradini, A. S. Cerqueira, A. Cerri, L. Cerrito, F. Cerutti, S. A. Cetin, A. Chafaq, D. Chakraborty, I. Chalupkova, K. Chan, P. Chang, B. Chapleau, J. D. Chapman, J. W. Chapman, D. G. Charlton, V. Chavda, C. A. Chavez Barajas, S. Cheatham, S. Chekanov, S. V. Chekulaev, G. A. Chelkov, M. A. Chelstowska, C. Chen, H. Chen, S. Chen, X. Chen, Y. Chen, Y. Cheng, A. Cheplakov, R. Cherkaoui El Moursli, V. Chernyatin, E. Cheu, S. L. Cheung, L. Chevalier, G. Chiefari, L. Chikovani, J. T. Childers, A. Chilingarov, G. Chiodini, A. S. Chisholm, R. T. Chislett, A. Chitan, M. V. Chizhov, G. Choudalakis, S. Chouridou, I. A. Christidi, A. Christov, D. Chromek-Burckhart, M. L. Chu, J. Chudoba, G. Ciapetti, A. K. Ciftci, R. Ciftci, D. Cinca, V. Cindro, A. Ciocio, M. Cirilli, P. Cirkovic, Z. H. Citron, M. Citterio, M. Ciubancan, A. Clark, P. J. Clark, R. N. Clarke, W. Cleland, J. C. Clemens, B. Clement, C. Clement, Y. Coadou, M. Cobal, A. Coccaro, J. Cochran, L. Coffey, J. G. Cogan, J. Coggeshall, J. Colas, S. Cole, A. P. Colijn, N. J. Collins, C. Collins-Tooth, J. Collot, T. Colombo, G. Colon, G. Compostella, P. Conde Muiño, E. Coniavitis, M. C. Conidi, S. M. Consonni, V. Consorti, S. Constantinescu, C. Conta, G. Conti, F. Conventi, M. Cooke, B. D. Cooper, A. M. Cooper-Sarkar, K. Copic, T. Cornelissen, M. Corradi, F. Corriveau, A. Cortes-Gonzalez, G. Cortiana, G. Costa, M. J. Costa, D. Costanzo, D. Côté, L. Courneyea, G. Cowan, B. E. Cox, K. Cranmer, F. Crescioli, M. Cristinziani, G. Crosetti, S. Crépé-Renaudin, C.-M. Cuciuc, C. Cuenca Almenar, T. Cuhadar Donszelmann, J. Cummings, M. Curatolo, C. J. Curtis, C. Cuthbert, P. Cwetanski, H. Czirr, P. Czodrowski, Z. Czyczula, S. D’Auria, M. D’Onofrio, A. D’Orazio, M. J. Da Cunha Sargedas De Sousa, C. Da Via, W. Dabrowski, A. Dafinca, T. Dai, F. Dallaire, C. Dallapiccola, M. Dam, M. Dameri, D. S. Damiani, H. O. Danielsson, V. Dao, G. Darbo, G. L. Darlea, J. A. Dassoulas, W. Davey, T. Davidek, N. Davidson, R. Davidson, E. Davies, M. Davies, O. Davignon, A. R. Davison, Y. Davygora, E. Dawe, I. Dawson, R. K. Daya-Ishmukhametova, K. De, R. de Asmundis, S. De Castro, S. De Cecco, J. de Graat, N. De Groot, P. de Jong, C. De La Taille, H. De la Torre, F. De Lorenzi, L. de Mora, L. De Nooij, D. De Pedis, A. De Salvo, U. De Sanctis, A. De Santo, J. B. De Vivie De Regie, G. De Zorzi, W. J. Dearnaley, R. Debbe, C. Debenedetti, B. Dechenaux, D. V. Dedovich, J. Degenhardt, J. Del Peso, T. Del Prete, T. Delemontex, M. Deliyergiyev, A. Dell’Acqua, L. Dell’Asta, M. Della Pietra, D. della Volpe, M. Delmastro, P. A. Delsart, C. Deluca, S. Demers, M. Demichev, B. Demirkoz, S. P. Denisov, D. Derendarz, J. E. Derkaoui, F. Derue, P. Dervan, K. Desch, E. Devetak, P. O. Deviveiros, A. Dewhurst, B. DeWilde, S. Dhaliwal, R. Dhullipudi, A. Di Ciaccio, L. Di Ciaccio, C. Di Donato, A. Di Girolamo, B. Di Girolamo, S. Di Luise, A. Di Mattia, B. Di Micco, R. Di Nardo, A. Di Simone, R. Di Sipio, M. A. Diaz, E. B. Diehl, J. Dietrich, T. A. Dietzsch, S. Diglio, K. Dindar Yagci, J. Dingfelder, F. Dinut, C. Dionisi, P. Dita, S. Dita, F. Dittus, F. Djama, T. Djobava, M. A. B. do Vale, A. Do Valle Wemans, T. K. O. Doan, M. Dobbs, D. Dobos, E. Dobson, J. Dodd, C. Doglioni, T. Doherty, Y. Doi, J. Dolejsi, Z. Dolezal, B. A. Dolgoshein, T. Dohmae, M. Donadelli, J. Donini, J. Dopke, A. Doria, A. Dos Anjos, A. Dotti, M. T. Dova, A. D. Doxiadis, A. T. Doyle, N. Dressnandt, M. Dris, J. Dubbert, S. Dube, E. Duchovni, G. Duckeck, D. Duda, A. Dudarev, F. Dudziak, M. Dührssen, I. P. Duerdoth, L. Duflot, M-A. Dufour, L. Duguid, M. Dunford, H. Duran Yildiz, R. Duxfield, M. Dwuznik, M. Düren, W. L. Ebenstein, J. Ebke, S. Eckweiler, W. Edson, C. A. Edwards, N. C. Edwards, W. Ehrenfeld, T. Eifert, G. Eigen, K. Einsweiler, E. Eisenhandler, T. Ekelof, M. El Kacimi, M. Ellert, S. Elles, F. Ellinghaus, K. Ellis, N. Ellis, J. Elmsheuser, M. Elsing, D. Emeliyanov, R. Engelmann, A. Engl, B. Epp, J. Erdmann, A. Ereditato, D. Eriksson, J. Ernst, M. Ernst, J. Ernwein, D. Errede, S. Errede, E. Ertel, M. Escalier, H. Esch, C. Escobar, X. Espinal Curull, B. Esposito, F. Etienne, A. I. Etienvre, E. Etzion, D. Evangelakou, H. Evans, L. Fabbri, C. Fabre, R. M. Fakhrutdinov, S. Falciano, Y. Fang, M. Fanti, A. Farbin, A. Farilla, J. Farley, T. Farooque, S. Farrell, S. M. Farrington, P. Farthouat, F. Fassi, P. Fassnacht, D. Fassouliotis, B. Fatholahzadeh, A. Favareto, L. Fayard, P. Federic, O. L. Fedin, W. Fedorko, M. Fehling-Kaschek, L. Feligioni, C. Feng, E. J. Feng, A. B. Fenyuk, J. Ferencei, W. Fernando, S. Ferrag, J. Ferrando, V. Ferrara, A. Ferrari, P. Ferrari, R. Ferrari, D. E. Ferreira de Lima, A. Ferrer, D. Ferrere, C. Ferretti, A. Ferretto Parodi, M. Fiascaris, F. Fiedler, A. Filipčič, F. Filthaut, M. Fincke-Keeler, M. C. N. Fiolhais, L. Fiorini, A. Firan, G. Fischer, M. J. Fisher, M. Flechl, I. Fleck, J. Fleckner, P. Fleischmann, S. Fleischmann, T. Flick, A. Floderus, L. R. Flores Castillo, A. C. Florez Bustos, M. J. Flowerdew, T. Fonseca Martin, A. Formica, A. Forti, D. Fortin, D. Fournier, A. J. Fowler, H. Fox, P. Francavilla, M. Franchini, S. Franchino, D. Francis, T. Frank, M. Franklin, S. Franz, M. Fraternali, S. Fratina, S. T. French, C. Friedrich, F. Friedrich, D. Froidevaux, J. A. Frost, C. Fukunaga, E. Fullana Torregrosa, B. G. Fulsom, J. Fuster, C. Gabaldon, O. Gabizon, T. Gadfort, S. Gadomski, G. Gagliardi, P. Gagnon, C. Galea, B. Galhardo, E. J. Gallas, V. Gallo, B. J. Gallop, P. Gallus, K. K. Gan, Y. S. Gao, A. Gaponenko, F. Garberson, M. Garcia-Sciveres, C. García, J. E. García Navarro, R. W. Gardner, N. Garelli, V. Garonne, C. Gatti, G. Gaudio, B. Gaur, L. Gauthier, P. Gauzzi, I. L. Gavrilenko, C. Gay, G. Gaycken, E. N. Gazis, P. Ge, Z. Gecse, C. N. P. Gee, D. A. A. Geerts, Ch. Geich-Gimbel, K. Gellerstedt, C. Gemme, A. Gemmell, M. H. Genest, S. Gentile, M. George, S. George, D. Gerbaudo, P. Gerlach, A. Gershon, C. Geweniger, H. Ghazlane, N. Ghodbane, B. Giacobbe, S. Giagu, V. Giangiobbe, F. Gianotti, B. Gibbard, A. Gibson, S. M. Gibson, M. Gilchriese, D. Gillberg, A. R. Gillman, D. M. Gingrich, J. Ginzburg, N. Giokaris, M. P. Giordani, R. Giordano, F. M. Giorgi, P. Giovannini, P. F. Giraud, D. Giugni, M. Giunta, B. K. Gjelsten, L. K. Gladilin, C. Glasman, J. Glatzer, A. Glazov, K. W. Glitza, G. L. Glonti, J. R. Goddard, J. Godfrey, J. Godlewski, M. Goebel, T. Göpfert, C. Goeringer, C. Gössling, S. Goldfarb, T. Golling, D. Golubkov, A. Gomes, L. S. Gomez Fajardo, R. Gonçalo, J. Goncalves Pinto Firmino Da Costa, L. Gonella, S. González de la Hoz, G. Gonzalez Parra, M. L. Gonzalez Silva, S. Gonzalez-Sevilla, J. J. Goodson, L. Goossens, P. A. Gorbounov, H. A. Gordon, I. Gorelov, G. Gorfine, B. Gorini, E. Gorini, A. Gorišek, E. Gornicki, A. T. Goshaw, M. Gosselink, M. I. Gostkin, I. Gough Eschrich, M. Gouighri, D. Goujdami, M. P. Goulette, A. G. Goussiou, C. Goy, S. Gozpinar, I. Grabowska-Bold, P. Grafström, K-J. Grahn, E. Gramstad, F. Grancagnolo, S. Grancagnolo, V. Grassi, V. Gratchev, H. M. Gray, J. A. Gray, E. Graziani, O. G. Grebenyuk, T. Greenshaw, Z. D. Greenwood, K. Gregersen, I. M. Gregor, P. Grenier, J. Griffiths, N. Grigalashvili, A. A. Grillo, K. Grimm, S. Grinstein, Ph. Gris, Y. V. Grishkevich, J.-F. Grivaz, A. Grohsjean, E. Gross, J. Grosse-Knetter, J. Groth-Jensen, K. Grybel, D. Guest, C. Guicheney, E. Guido, S. Guindon, U. Gul, J. Gunther, B. Guo, J. Guo, P. Gutierrez, N. Guttman, O. Gutzwiller, C. Guyot, C. Gwenlan, C. B. Gwilliam, A. Haas, S. Haas, C. Haber, H. K. Hadavand, D. R. Hadley, P. Haefner, F. Hahn, Z. Hajduk, H. Hakobyan, D. Hall, K. Hamacher, P. Hamal, K. Hamano, M. Hamer, A. Hamilton, S. Hamilton, L. Han, K. Hanagaki, K. Hanawa, M. Hance, C. Handel, P. Hanke, J. R. Hansen, J. B. Hansen, J. D. Hansen, P. H. Hansen, P. Hansson, K. Hara, T. Harenberg, S. Harkusha, D. Harper, R. D. Harrington, O. M. Harris, J. Hartert, F. Hartjes, T. Haruyama, A. Harvey, S. Hasegawa, Y. Hasegawa, S. Hassani, S. Haug, M. Hauschild, R. Hauser, M. Havranek, C. M. Hawkes, R. J. Hawkings, A. D. Hawkins, T. Hayakawa, T. Hayashi, D. Hayden, C. P. Hays, H. S. Hayward, S. J. Haywood, S. J. Head, V. Hedberg, L. Heelan, S. Heim, B. Heinemann, S. Heisterkamp, L. Helary, C. Heller, M. Heller, S. Hellman, D. Hellmich, C. Helsens, R. C. W. Henderson, M. Henke, A. Henrichs, A. M. Henriques Correia, S. Henrot-Versille, C. Hensel, C. M. Hernandez, Y. Hernández Jiménez, R. Herrberg, G. Herten, R. Hertenberger, L. Hervas, G. G. Hesketh, N. P. Hessey, E. Higón-Rodriguez, J. C. Hill, K. H. Hiller, S. Hillert, S. J. Hillier, I. Hinchliffe, E. Hines, M. Hirose, F. Hirsch, D. Hirschbuehl, J. Hobbs, N. Hod, M. C. Hodgkinson, P. Hodgson, A. Hoecker, M. R. Hoeferkamp, J. Hoffman, D. Hoffmann, M. Hohlfeld, M. Holder, S. O. Holmgren, T. Holy, J. L. Holzbauer, T. M. Hong, L. Hooft van Huysduynen, S. Horner, J-Y. Hostachy, S. Hou, A. Hoummada, J. Howard, J. Howarth, I. Hristova, J. Hrivnac, T. Hryn’ova, P. J. Hsu, S.-C. Hsu, D. Hu, Z. Hubacek, F. Hubaut, F. Huegging, A. Huettmann, T. B. Huffman, E. W. Hughes, G. Hughes, M. Huhtinen, M. Hurwitz, N. Huseynov, J. Huston, J. Huth, G. Iacobucci, G. Iakovidis, M. Ibbotson, I. Ibragimov, L. Iconomidou-Fayard, J. Idarraga, P. Iengo, O. Igonkina, Y. Ikegami, M. Ikeno, D. Iliadis, N. Ilic, T. Ince, P. Ioannou, M. Iodice, K. Iordanidou, V. Ippolito, A. Irles Quiles, C. Isaksson, M. Ishino, M. Ishitsuka, R. Ishmukhametov, C. Issever, S. Istin, A. V. Ivashin, W. Iwanski, H. Iwasaki, J. M. Izen, V. Izzo, B. Jackson, J. N. Jackson, P. Jackson, M. R. Jaekel, V. Jain, K. Jakobs, S. Jakobsen, T. Jakoubek, J. Jakubek, D. O. Jamin, D. K. Jana, E. Jansen, H. Jansen, J. Janssen, A. Jantsch, M. Janus, R. C. Jared, G. Jarlskog, L. Jeanty, I. Jen-La Plante, G.-Y. Jeng, D. Jennens, P. Jenni, A. E. Loevschall-Jensen, P. Jež, S. Jézéquel, M. K. Jha, H. Ji, W. Ji, J. Jia, Y. Jiang, M. Jimenez Belenguer, S. Jin, O. Jinnouchi, M. D. Joergensen, D. Joffe, M. Johansen, K. E. Johansson, P. Johansson, S. Johnert, K. A. Johns, K. Jon-And, G. Jones, R. W. L. Jones, T. J. Jones, C. Joram, P. M. Jorge, K. D. Joshi, J. Jovicevic, T. Jovin, X. Ju, C. A. Jung, R. M. Jungst, V. Juranek, P. Jussel, A. Juste Rozas, S. Kabana, M. Kaci, A. Kaczmarska, P. Kadlecik, M. Kado, H. Kagan, M. Kagan, E. Kajomovitz, S. Kalinin, L. V. Kalinovskaya, S. Kama, N. Kanaya, M. Kaneda, S. Kaneti, T. Kanno, V. A. Kantserov, J. Kanzaki, B. Kaplan, A. Kapliy, D. Kar, M. Karagounis, K. Karakostas, M. Karnevskiy, V. Kartvelishvili, A. N. Karyukhin, L. Kashif, G. Kasieczka, R. D. Kass, A. Kastanas, M. Kataoka, Y. Kataoka, J. Katzy, V. Kaushik, K. Kawagoe, T. Kawamoto, G. Kawamura, M. S. Kayl, S. Kazama, V. F. Kazanin, M. Y. Kazarinov, R. Keeler, P. T. Keener, R. Kehoe, M. Keil, G. D. Kekelidze, J. S. Keller, M. Kenyon, O. Kepka, N. Kerschen, B. P. Kerševan, S. Kersten, K. Kessoku, J. Keung, F. Khalil-zada, H. Khandanyan, A. Khanov, D. Kharchenko, A. Khodinov, A. Khomich, T. J. Khoo, G. Khoriauli, A. Khoroshilov, V. Khovanskiy, E. Khramov, J. Khubua, H. Kim, S. H. Kim, N. Kimura, O. Kind, B. T. King, M. King, R. S. B. King, J. Kirk, A. E. Kiryunin, T. Kishimoto, D. Kisielewska, T. Kitamura, T. Kittelmann, K. Kiuchi, E. Kladiva, M. Klein, U. Klein, K. Kleinknecht, M. Klemetti, A. Klier, P. Klimek, A. Klimentov, R. Klingenberg, J. A. Klinger, E. B. Klinkby, T. Klioutchnikova, P. F. Klok, S. Klous, E.-E. Kluge, T. Kluge, P. Kluit, S. Kluth, E. Kneringer, E. B. F. G. Knoops, A. Knue, B. R. Ko, T. Kobayashi, M. Kobel, M. Kocian, P. Kodys, K. Köneke, A. C. König, S. Koenig, L. Köpke, F. Koetsveld, P. Koevesarki, T. Koffas, E. Koffeman, L. A. Kogan, S. Kohlmann, F. Kohn, Z. Kohout, T. Kohriki, T. Koi, G. M. Kolachev, H. Kolanoski, V. Kolesnikov, I. Koletsou, J. Koll, A. A. Komar, Y. Komori, T. Kondo, T. Kono, A. I. Kononov, R. Konoplich, N. Konstantinidis, R. Kopeliansky, S. Koperny, K. Korcyl, K. Kordas, A. Korn, A. Korol, I. Korolkov, E. V. Korolkova, V. A. Korotkov, O. Kortner, S. Kortner, V. V. Kostyukhin, S. Kotov, V. M. Kotov, A. Kotwal, C. Kourkoumelis, V. Kouskoura, A. Koutsman, R. Kowalewski, T. Z. Kowalski, W. Kozanecki, A. S. Kozhin, V. Kral, V. A. Kramarenko, G. Kramberger, M. W. Krasny, A. Krasznahorkay, J. K. Kraus, A. Kravchenko, S. Kreiss, F. Krejci, J. Kretzschmar, K. Kreutzfeldt, N. Krieger, P. Krieger, K. Kroeninger, H. Kroha, J. Kroll, J. Kroseberg, J. Krstic, U. Kruchonak, H. Krüger, T. Kruker, N. Krumnack, Z. V. Krumshteyn, M. K. Kruse, T. Kubota, S. Kuday, S. Kuehn, A. Kugel, T. Kuhl, D. Kuhn, V. Kukhtin, Y. Kulchitsky, S. Kuleshov, C. Kummer, M. Kuna, J. Kunkle, A. Kupco, H. Kurashige, M. Kurata, Y. A. Kurochkin, V. Kus, E. S. Kuwertz, M. Kuze, J. Kvita, R. Kwee, A. La Rosa, L. La Rotonda, L. Labarga, S. Lablak, C. Lacasta, F. Lacava, J. Lacey, H. Lacker, D. Lacour, V. R. Lacuesta, E. Ladygin, R. Lafaye, B. Laforge, T. Lagouri, S. Lai, E. Laisne, L. Lambourne, C. L. Lampen, W. Lampl, E. Lancon, U. Landgraf, M. P. J. Landon, V. S. Lang, C. Lange, A. J. Lankford, F. Lanni, K. Lantzsch, A. Lanza, S. Laplace, C. Lapoire, J. F. Laporte, T. Lari, A. Larner, M. Lassnig, P. Laurelli, V. Lavorini, W. Lavrijsen, P. Laycock, O. Le Dortz, E. Le Guirriec, E. Le Menedeu, T. LeCompte, F. Ledroit-Guillon, H. Lee, J. S. H. Lee, S. C. Lee, L. Lee, M. Lefebvre, M. Legendre, F. Legger, C. Leggett, M. Lehmacher, G. Lehmann Miotto, A. G. Leister, M. A. L. Leite, R. Leitner, D. Lellouch, B. Lemmer, V. Lendermann, K. J. C. Leney, T. Lenz, G. Lenzen, B. Lenzi, K. Leonhardt, S. Leontsinis, F. Lepold, C. Leroy, J-R. Lessard, C. G. Lester, C. M. Lester, J. Levêque, D. Levin, L. J. Levinson, A. Lewis, G. H. Lewis, A. M. Leyko, M. Leyton, B. Li, B. Li, H. Li, H. L. Li, S. Li, X. Li, Z. Liang, H. Liao, B. Liberti, P. Lichard, M. Lichtnecker, K. Lie, W. Liebig, C. Limbach, A. Limosani, M. Limper, S. C. Lin, F. Linde, J. T. Linnemann, E. Lipeles, A. Lipniacka, T. M. Liss, D. Lissauer, A. Lister, A. M. Litke, C. Liu, D. Liu, J. B. Liu, L. Liu, M. Liu, Y. Liu, M. Livan, S. S. A. Livermore, A. Lleres, J. Llorente Merino, S. L. Lloyd, E. Lobodzinska, P. Loch, W. S. Lockman, T. Loddenkoetter, F. K. Loebinger, A. Loginov, C. W. Loh, T. Lohse, K. Lohwasser, M. Lokajicek, V. P. Lombardo, R. E. Long, L. Lopes, D. Lopez Mateos, J. Lorenz, N. Lorenzo Martinez, M. Losada, P. Loscutoff, F. Lo Sterzo, M. J. Losty, X. Lou, A. Lounis, K. F. Loureiro, J. Love, P. A. Love, A. J. Lowe, F. Lu, H. J. Lubatti, C. Luci, A. Lucotte, D. Ludwig, I. Ludwig, J. Ludwig, F. Luehring, G. Luijckx, W. Lukas, L. Luminari, E. Lund, B. Lund-Jensen, B. Lundberg, J. Lundberg, O. Lundberg, J. Lundquist, M. Lungwitz, D. Lynn, E. Lytken, H. Ma, L. L. Ma, G. Maccarrone, A. Macchiolo, B. Maček, J. Machado Miguens, D. Macina, R. Mackeprang, R. J. Madaras, H. J. Maddocks, W. F. Mader, R. Maenner, T. Maeno, P. Mättig, S. Mättig, L. Magnoni, E. Magradze, K. Mahboubi, J. Mahlstedt, S. Mahmoud, G. Mahout, C. Maiani, C. Maidantchik, A. Maio, S. Majewski, Y. Makida, N. Makovec, P. Mal, B. Malaescu, Pa. Malecki, P. Malecki, V. P. Maleev, F. Malek, U. Mallik, D. Malon, C. Malone, S. Maltezos, V. Malyshev, S. Malyukov, J. Mamuzic, A. Manabe, L. Mandelli, I. Mandić, R. Mandrysch, J. Maneira, A. Manfredini, L. Manhaes de Andrade Filho, J. A. Manjarres Ramos, A. Mann, P. M. Manning, A. Manousakis-Katsikakis, B. Mansoulie, R. Mantifel, A. Mapelli, L. Mapelli, L. March, J. F. Marchand, F. Marchese, G. Marchiori, M. Marcisovsky, C. P. Marino, F. Marroquim, Z. Marshall, L. F. Marti, S. Marti-Garcia, B. Martin, B. Martin, J. P. Martin, T. A. Martin, V. J. Martin, B. Martin dit Latour, S. Martin-Haugh, H. Martinez, M. Martinez, V. Martinez Outschoorn, A. C. Martyniuk, M. Marx, F. Marzano, A. Marzin, L. Masetti, T. Mashimo, R. Mashinistov, J. Masik, A. L. Maslennikov, I. Massa, G. Massaro, N. Massol, P. Mastrandrea, A. Mastroberardino, T. Masubuchi, H. Matsunaga, T. Matsushita, C. Mattravers, J. Maurer, S. J. Maxfield, D. A. Maximov, A. Mayne, R. Mazini, M. Mazur, L. Mazzaferro, M. Mazzanti, J. Mc Donald, S. P. Mc Kee, A. McCarn, R. L. McCarthy, T. G. McCarthy, N. A. McCubbin, K. W. McFarlane, J. A. Mcfayden, G. Mchedlidze, T. Mclaughlan, S. J. McMahon, R. A. McPherson, A. Meade, J. Mechnich, M. Mechtel, M. Medinnis, S. Meehan, R. Meera-Lebbai, T. Meguro, S. Mehlhase, A. Mehta, K. Meier, B. Meirose, C. Melachrinos, B. R. Mellado Garcia, F. Meloni, L. Mendoza Navas, Z. Meng, A. Mengarelli, S. Menke, E. Meoni, K. M. Mercurio, P. Mermod, L. Merola, C. Meroni, F. S. Merritt, H. Merritt, A. Messina, J. Metcalfe, A. S. Mete, C. Meyer, C. Meyer, J-P. Meyer, J. Meyer, J. Meyer, S. Michal, L. Micu, R. P. Middleton, S. Migas, L. Mijović, G. Mikenberg, M. Mikestikova, M. Mikuž, D. W. Miller, R. J. Miller, W. J. Mills, C. Mills, A. Milov, D. A. Milstead, D. Milstein, A. A. Minaenko, M. Miñano Moya, I. A. Minashvili, A. I. Mincer, B. Mindur, M. Mineev, Y. Ming, L. M. Mir, G. Mirabelli, J. Mitrevski, V. A. Mitsou, S. Mitsui, P. S. Miyagawa, J. U. Mjörnmark, T. Moa, V. Moeller, K. Mönig, N. Möser, S. Mohapatra, W. Mohr, R. Moles-Valls, A. Molfetas, J. Monk, E. Monnier, J. Montejo Berlingen, F. Monticelli, S. Monzani, R. W. Moore, G. F. Moorhead, C. Mora Herrera, A. Moraes, N. Morange, J. Morel, G. Morello, D. Moreno, M. Moreno Llácer, P. Morettini, M. Morgenstern, M. Morii, A. K. Morley, G. Mornacchi, J. D. Morris, L. Morvaj, H. G. Moser, M. Mosidze, J. Moss, R. Mount, E. Mountricha, S. V. Mouraviev, E. J. W. Moyse, F. Mueller, J. Mueller, K. Mueller, T. A. Müller, T. Mueller, D. Muenstermann, Y. Munwes, W. J. Murray, I. Mussche, E. Musto, A. G. Myagkov, M. Myska, O. Nackenhorst, J. Nadal, K. Nagai, R. Nagai, K. Nagano, A. Nagarkar, Y. Nagasaka, M. Nagel, A. M. Nairz, Y. Nakahama, K. Nakamura, T. Nakamura, I. Nakano, G. Nanava, A. Napier, R. Narayan, M. Nash, T. Nattermann, T. Naumann, G. Navarro, H. A. Neal, P. Yu. Nechaeva, T. J. Neep, A. Negri, G. Negri, M. Negrini, S. Nektarijevic, A. Nelson, T. K. Nelson, S. Nemecek, P. Nemethy, A. A. Nepomuceno, M. Nessi, M. S. Neubauer, M. Neumann, A. Neusiedl, R. M. Neves, P. Nevski, F. M. Newcomer, P. R. Newman, V. Nguyen Thi Hong, R. B. Nickerson, R. Nicolaidou, B. Nicquevert, F. Niedercorn, J. Nielsen, N. Nikiforou, A. Nikiforov, V. Nikolaenko, I. Nikolic-Audit, K. Nikolics, K. Nikolopoulos, H. Nilsen, P. Nilsson, Y. Ninomiya, A. Nisati, R. Nisius, T. Nobe, L. Nodulman, M. Nomachi, I. Nomidis, S. Norberg, M. Nordberg, J. Novakova, M. Nozaki, L. Nozka, A.-E. Nuncio-Quiroz, G. Nunes Hanninger, T. Nunnemann, E. Nurse, B. J. O’Brien, D. C. O’Neil, V. O’Shea, L. B. Oakes, F. G. Oakham, H. Oberlack, J. Ocariz, A. Ochi, S. Oda, S. Odaka, J. Odier, H. Ogren, A. Oh, S. H. Oh, C. C. Ohm, T. Ohshima, W. Okamura, H. Okawa, Y. Okumura, T. Okuyama, A. Olariu, A. G. Olchevski, S. A. Olivares Pino, M. Oliveira, D. Oliveira Damazio, E. Oliver Garcia, D. Olivito, A. Olszewski, J. Olszowska, A. Onofre, P. U. E. Onyisi, C. J. Oram, M. J. Oreglia, Y. Oren, D. Orestano, N. Orlando, I. Orlov, C. Oropeza Barrera, R. S. Orr, B. Osculati, R. Ospanov, C. Osuna, G. Otero y Garzon, J. P. Ottersbach, M. Ouchrif, E. A. Ouellette, F. Ould-Saada, A. Ouraou, Q. Ouyang, A. Ovcharova, M. Owen, S. Owen, V. E. Ozcan, N. Ozturk, A. Pacheco Pages, C. Padilla Aranda, S. Pagan Griso, E. Paganis, C. Pahl, F. Paige, P. Pais, K. Pajchel, G. Palacino, C. P. Paleari, S. Palestini, D. Pallin, A. Palma, J. D. Palmer, Y. B. Pan, E. Panagiotopoulou, J. G. Panduro Vazquez, P. Pani, N. Panikashvili, S. Panitkin, D. Pantea, A. Papadelis, Th. D. Papadopoulou, A. Paramonov, D. Paredes Hernandez, W. Park, M. A. Parker, F. Parodi, J. A. Parsons, U. Parzefall, S. Pashapour, E. Pasqualucci, S. Passaggio, A. Passeri, F. Pastore, Fr. Pastore, G. Pásztor, S. Pataraia, N. Patel, J. R. Pater, S. Patricelli, T. Pauly, M. Pecsy, S. Pedraza Lopez, M. I. Pedraza Morales, S. V. Peleganchuk, D. Pelikan, H. Peng, B. Penning, A. Penson, J. Penwell, M. Perantoni, K. Perez, T. Perez Cavalcanti, E. Perez Codina, M. T. Pérez García-Estañ, V. Perez Reale, L. Perini, H. Pernegger, R. Perrino, P. Perrodo, V. D. Peshekhonov, K. Peters, B. A. Petersen, J. Petersen, T. C. Petersen, E. Petit, A. Petridis, C. Petridou, E. Petrolo, F. Petrucci, D. Petschull, M. Petteni, R. Pezoa, A. Phan, P. W. Phillips, G. Piacquadio, A. Picazio, E. Piccaro, M. Piccinini, S. M. Piec, R. Piegaia, D. T. Pignotti, J. E. Pilcher, A. D. Pilkington, J. Pina, M. Pinamonti, A. Pinder, J. L. Pinfold, A. Pingel, B. Pinto, C. Pizio, M.-A. Pleier, E. Plotnikova, A. Poblaguev, S. Poddar, F. Podlyski, L. Poggioli, D. Pohl, M. Pohl, G. Polesello, A. Policicchio, A. Polini, J. Poll, V. Polychronakos, D. Pomeroy, K. Pommès, L. Pontecorvo, B. G. Pope, G. A. Popeneciu, D. S. Popovic, A. Poppleton, X. Portell Bueso, G. E. Pospelov, S. Pospisil, I. N. Potrap, C. J. Potter, C. T. Potter, G. Poulard, J. Poveda, V. Pozdnyakov, R. Prabhu, P. Pralavorio, A. Pranko, S. Prasad, R. Pravahan, S. Prell, K. Pretzl, D. Price, J. Price, L. E. Price, D. Prieur, M. Primavera, K. Prokofiev, F. Prokoshin, S. Protopopescu, J. Proudfoot, X. Prudent, M. Przybycien, H. Przysiezniak, S. Psoroulas, E. Ptacek, E. Pueschel, D. Puldon, J. Purdham, M. Purohit, P. Puzo, Y. Pylypchenko, J. Qian, A. Quadt, D. R. Quarrie, W. B. Quayle, M. Raas, V. Radeka, V. Radescu, P. Radloff, F. Ragusa, G. Rahal, A. M. Rahimi, D. Rahm, S. Rajagopalan, M. Rammensee, M. Rammes, A. S. Randle-Conde, K. Randrianarivony, K. Rao, F. Rauscher, T. C. Rave, M. Raymond, A. L. Read, D. M. Rebuzzi, A. Redelbach, G. Redlinger, R. Reece, K. Reeves, A. Reinsch, I. Reisinger, C. Rembser, Z. L. Ren, A. Renaud, M. Rescigno, S. Resconi, B. Resende, P. Reznicek, R. Rezvani, R. Richter, E. Richter-Was, M. Ridel, M. Rijpstra, M. Rijssenbeek, A. Rimoldi, L. Rinaldi, R. R. Rios, I. Riu, G. Rivoltella, F. Rizatdinova, E. Rizvi, S. H. Robertson, A. Robichaud-Veronneau, D. Robinson, J. E. M. Robinson, A. Robson, J. G. Rocha de Lima, C. Roda, D. Roda Dos Santos, A. Roe, S. Roe, O. Røhne, S. Rolli, A. Romaniouk, M. Romano, G. Romeo, E. Romero Adam, N. Rompotis, L. Roos, E. Ros, S. Rosati, K. Rosbach, A. Rose, M. Rose, G. A. Rosenbaum, P. L. Rosendahl, O. Rosenthal, L. Rosselet, V. Rossetti, E. Rossi, L. P. Rossi, M. Rotaru, I. Roth, J. Rothberg, D. Rousseau, C. R. Royon, A. Rozanov, Y. Rozen, X. Ruan, F. Rubbo, I. Rubinskiy, N. Ruckstuhl, V. I. Rud, C. Rudolph, G. Rudolph, F. Rühr, A. Ruiz-Martinez, L. Rumyantsev, Z. Rurikova, N. A. Rusakovich, A. Ruschke, J. P. Rutherfoord, N. Ruthmann, P. Ruzicka, Y. F. Ryabov, M. Rybar, G. Rybkin, N. C. Ryder, A. F. Saavedra, I. Sadeh, H. F-W. Sadrozinski, R. Sadykov, F. Safai Tehrani, H. Sakamoto, G. Salamanna, A. Salamon, M. Saleem, D. Salek, D. Salihagic, A. Salnikov, J. Salt, B. M. Salvachua Ferrando, D. Salvatore, F. Salvatore, A. Salvucci, A. Salzburger, D. Sampsonidis, B. H. Samset, A. Sanchez, V. Sanchez Martinez, H. Sandaker, H. G. Sander, M. P. Sanders, M. Sandhoff, T. Sandoval, C. Sandoval, R. Sandstroem, D. P. C. Sankey, A. Sansoni, C. Santamarina Rios, C. Santoni, R. Santonico, H. Santos, I. Santoyo Castillo, J. G. Saraiva, T. Sarangi, E. Sarkisyan-Grinbaum, B. Sarrazin, F. Sarri, G. Sartisohn, O. Sasaki, Y. Sasaki, N. Sasao, I. Satsounkevitch, G. Sauvage, E. Sauvan, J. B. Sauvan, P. Savard, V. Savinov, D. O. Savu, L. Sawyer, D. H. Saxon, J. Saxon, C. Sbarra, A. Sbrizzi, D. A. Scannicchio, M. Scarcella, J. Schaarschmidt, P. Schacht, D. Schaefer, U. Schäfer, A. Schaelicke, S. Schaepe, S. Schaetzel, A. C. Schaffer, D. Schaile, R. D. Schamberger, A. G. Schamov, V. Scharf, V. A. Schegelsky, D. Scheirich, M. Schernau, M. I. Scherzer, C. Schiavi, J. Schieck, M. Schioppa, S. Schlenker, E. Schmidt, K. Schmieden, C. Schmitt, S. Schmitt, B. Schneider, U. Schnoor, L. Schoeffel, A. Schoening, A. L. S. Schorlemmer, M. Schott, D. Schouten, J. Schovancova, M. Schram, C. Schroeder, N. Schroer, M. J. Schultens, J. Schultes, H.-C. Schultz-Coulon, H. Schulz, M. Schumacher, B. A. Schumm, Ph. Schune, A. Schwartzman, Ph. Schwegler, Ph. Schwemling, R. Schwienhorst, R. Schwierz, J. Schwindling, T. Schwindt, M. Schwoerer, F. G. Sciacca, G. Sciolla, W. G. Scott, J. Searcy, G. Sedov, E. Sedykh, S. C. Seidel, A. Seiden, F. Seifert, J. M. Seixas, G. Sekhniaidze, S. J. Sekula, K. E. Selbach, D. M. Seliverstov, B. Sellden, G. Sellers, M. Seman, N. Semprini-Cesari, C. Serfon, L. Serin, L. Serkin, R. Seuster, H. Severini, A. Sfyrla, E. Shabalina, M. Shamim, L. Y. Shan, J. T. Shank, Q. T. Shao, M. Shapiro, P. B. Shatalov, K. Shaw, D. Sherman, P. Sherwood, S. Shimizu, M. Shimojima, T. Shin, M. Shiyakova, A. Shmeleva, M. J. Shochet, D. Short, S. Shrestha, E. Shulga, M. A. Shupe, P. Sicho, A. Sidoti, F. Siegert, Dj. Sijacki, O. Silbert, J. Silva, Y. Silver, D. Silverstein, S. B. Silverstein, V. Simak, O. Simard, Lj. Simic, S. Simion, E. Simioni, B. Simmons, R. Simoniello, M. Simonyan, P. Sinervo, N. B. Sinev, V. Sipica, G. Siragusa, A. Sircar, A. N. Sisakyan, S. Yu. Sivoklokov, J. Sjölin, T. B. Sjursen, L. A. Skinnari, H. P. Skottowe, K. Skovpen, P. Skubic, M. Slater, T. Slavicek, K. Sliwa, V. Smakhtin, B. H. Smart, L. Smestad, S. Yu. Smirnov, Y. Smirnov, L. N. Smirnova, O. Smirnova, B. C. Smith, D. Smith, K. M. Smith, M. Smizanska, K. Smolek, A. A. Snesarev, S. W. Snow, J. Snow, S. Snyder, R. Sobie, J. Sodomka, A. Soffer, C. A. Solans, M. Solar, J. Solc, E. Yu. Soldatov, U. Soldevila, E. Solfaroli Camillocci, A. A. Solodkov, O. V. Solovyanov, V. Solovyev, N. Soni, A. Sood, V. Sopko, B. Sopko, M. Sosebee, R. Soualah, P. Soueid, A. Soukharev, S. Spagnolo, F. Spanò, R. Spighi, G. Spigo, R. Spiwoks, M. Spousta, T. Spreitzer, B. Spurlock, R. D. St. Denis, J. Stahlman, R. Stamen, E. Stanecka, R. W. Stanek, C. Stanescu, M. Stanescu-Bellu, M. M. Stanitzki, S. Stapnes, E. A. Starchenko, J. Stark, P. Staroba, P. Starovoitov, R. Staszewski, A. Staude, P. Stavina, G. Steele, P. Steinbach, P. Steinberg, I. Stekl, B. Stelzer, H. J. Stelzer, O. Stelzer-Chilton, H. Stenzel, S. Stern, G. A. Stewart, J. A. Stillings, M. C. Stockton, M. Stoebe, K. Stoerig, G. Stoicea, S. Stonjek, P. Strachota, A. R. Stradling, A. Straessner, J. Strandberg, S. Strandberg, A. Strandlie, M. Strang, E. Strauss, M. Strauss, P. Strizenec, R. Ströhmer, D. M. Strom, J. A. Strong, R. Stroynowski, B. Stugu, I. Stumer, J. Stupak, P. Sturm, N. A. Styles, D. A. Soh, D. Su, HS. Subramania, R. Subramaniam, A. Succurro, Y. Sugaya, C. Suhr, M. Suk, V. V. Sulin, S. Sultansoy, T. Sumida, X. Sun, J. E. Sundermann, K. Suruliz, G. Susinno, M. R. Sutton, Y. Suzuki, Y. Suzuki, M. Svatos, S. Swedish, I. Sykora, T. Sykora, J. Sánchez, D. Ta, K. Tackmann, A. Taffard, R. Tafirout, N. Taiblum, Y. Takahashi, H. Takai, R. Takashima, H. Takeda, T. Takeshita, Y. Takubo, M. Talby, A. Talyshev, M. C. Tamsett, K. G. Tan, J. Tanaka, R. Tanaka, S. Tanaka, S. Tanaka, A. J. Tanasijczuk, K. Tani, N. Tannoury, S. Tapprogge, D. Tardif, S. Tarem, F. Tarrade, G. F. Tartarelli, P. Tas, M. Tasevsky, E. Tassi, Y. Tayalati, C. Taylor, F. E. Taylor, G. N. Taylor, W. Taylor, M. Teinturier, F. A. Teischinger, M. Teixeira Dias Castanheira, P. Teixeira-Dias, K. K. Temming, H. Ten Kate, P. K. Teng, S. Terada, K. Terashi, J. Terron, M. Testa, R. J. Teuscher, J. Therhaag, T. Theveneaux-Pelzer, S. Thoma, J. P. Thomas, E. N. Thompson, P. D. Thompson, P. D. Thompson, A. S. Thompson, L. A. Thomsen, E. Thomson, M. Thomson, W. M. Thong, R. P. Thun, F. Tian, M. J. Tibbetts, T. Tic, V. O. Tikhomirov, Y. A. Tikhonov, S. Timoshenko, E. Tiouchichine, P. Tipton, S. Tisserant, T. Todorov, S. Todorova-Nova, B. Toggerson, J. Tojo, S. Tokár, K. Tokushuku, K. Tollefson, M. Tomoto, L. Tompkins, K. Toms, A. Tonoyan, C. Topfel, N. D. Topilin, E. Torrence, H. Torres, E. Torró Pastor, J. Toth, F. Touchard, D. R. Tovey, T. Trefzger, L. Tremblet, A. Tricoli, I. M. Trigger, S. Trincaz-Duvoid, M. F. Tripiana, N. Triplett, W. Trischuk, B. Trocmé, C. Troncon, M. Trottier-McDonald, P. True, M. Trzebinski, A. Trzupek, C. Tsarouchas, J. C-L. Tseng, M. Tsiakiris, P. V. Tsiareshka, D. Tsionou, G. Tsipolitis, S. Tsiskaridze, V. Tsiskaridze, E. G. Tskhadadze, I. I. Tsukerman, V. Tsulaia, J.-W. Tsung, S. Tsuno, D. Tsybychev, A. Tua, A. Tudorache, V. Tudorache, J. M. Tuggle, M. Turala, D. Turecek, I. Turk Cakir, E. Turlay, R. Turra, P. M. Tuts, A. Tykhonov, M. Tylmad, M. Tyndel, G. Tzanakos, K. Uchida, I. Ueda, R. Ueno, M. Ughetto, M. Ugland, M. Uhlenbrock, M. Uhrmacher, F. Ukegawa, G. Unal, A. Undrus, G. Unel, Y. Unno, D. Urbaniec, P. Urquijo, G. Usai, M. Uslenghi, L. Vacavant, V. Vacek, B. Vachon, S. Vahsen, S. Valentinetti, A. Valero, S. Valkar, E. Valladolid Gallego, S. Vallecorsa, J. A. Valls Ferrer, R. Van Berg, P. C. Van Der Deijl, R. van der Geer, H. van der Graaf, R. Van Der Leeuw, E. van der Poel, D. van der Ster, N. van Eldik, P. van Gemmeren, J. Van Nieuwkoop, I. van Vulpen, M. Vanadia, W. Vandelli, A. Vaniachine, P. Vankov, F. Vannucci, R. Vari, E. W. Varnes, T. Varol, D. Varouchas, A. Vartapetian, K. E. Varvell, V. I. Vassilakopoulos, F. Vazeille, T. Vazquez Schroeder, G. Vegni, J. J. Veillet, F. Veloso, R. Veness, S. Veneziano, A. Ventura, D. Ventura, M. Venturi, N. Venturi, V. Vercesi, M. Verducci, W. Verkerke, J. C. Vermeulen, A. Vest, M. C. Vetterli, I. Vichou, T. Vickey, O. E. Vickey Boeriu, G. H. A. Viehhauser, S. Viel, M. Villa, M. Villaplana Perez, E. Vilucchi, M. G. Vincter, E. Vinek, V. B. Vinogradov, M. Virchaux, J. Virzi, O. Vitells, M. Viti, I. Vivarelli, F. Vives Vaque, S. Vlachos, D. Vladoiu, M. Vlasak, A. Vogel, P. Vokac, G. Volpi, M. Volpi, G. Volpini, H. von der Schmitt, H. von Radziewski, E. von Toerne, V. Vorobel, V. Vorwerk, M. Vos, R. Voss, J. H. Vossebeld, N. Vranjes, M. Vranjes Milosavljevic, V. Vrba, M. Vreeswijk, T. Vu Anh, R. Vuillermet, I. Vukotic, W. Wagner, P. Wagner, H. Wahlen, S. Wahrmund, J. Wakabayashi, S. Walch, J. Walder, R. Walker, W. Walkowiak, R. Wall, P. Waller, B. Walsh, C. Wang, H. Wang, H. Wang, J. Wang, J. Wang, R. Wang, S. M. Wang, T. Wang, A. Warburton, C. P. Ward, D. R. Wardrope, M. Warsinsky, A. Washbrook, C. Wasicki, I. Watanabe, P. M. Watkins, A. T. Watson, I. J. Watson, M. F. Watson, G. Watts, S. Watts, A. T. Waugh, B. M. Waugh, M. S. Weber, J. S. Webster, A. R. Weidberg, P. Weigell, J. Weingarten, C. Weiser, P. S. Wells, T. Wenaus, D. Wendland, Z. Weng, T. Wengler, S. Wenig, N. Wermes, M. Werner, P. Werner, M. Werth, M. Wessels, J. Wetter, C. Weydert, K. Whalen, A. White, M. J. White, S. White, S. R. Whitehead, D. Whiteson, D. Whittington, D. Wicke, F. J. Wickens, W. Wiedenmann, M. Wielers, P. Wienemann, C. Wiglesworth, L. A. M. Wiik-Fuchs, P. A. Wijeratne, A. Wildauer, M. A. Wildt, I. Wilhelm, H. G. Wilkens, J. Z. Will, E. Williams, H. H. Williams, S. Williams, W. Willis, S. Willocq, J. A. Wilson, M. G. Wilson, A. Wilson, I. Wingerter-Seez, S. Winkelmann, F. Winklmeier, M. Wittgen, S. J. Wollstadt, M. W. Wolter, H. Wolters, W. C. Wong, G. Wooden, B. K. Wosiek, J. Wotschack, M. J. Woudstra, K. W. Wozniak, K. Wraight, M. Wright, B. Wrona, S. L. Wu, X. Wu, Y. Wu, E. Wulf, B. M. Wynne, S. Xella, M. Xiao, S. Xie, C. Xu, D. Xu, L. Xu, B. Yabsley, S. Yacoob, M. Yamada, H. Yamaguchi, A. Yamamoto, K. Yamamoto, S. Yamamoto, T. Yamamura, T. Yamanaka, K. Yamauchi, T. Yamazaki, Y. Yamazaki, Z. Yan, H. Yang, U. K. Yang, Y. Yang, Z. Yang, S. Yanush, L. Yao, Y. Yasu, E. Yatsenko, J. Ye, S. Ye, A. L. Yen, M. Yilmaz, R. Yoosoofmiya, K. Yorita, R. Yoshida, K. Yoshihara, C. Young, C. J. Young, S. Youssef, D. Yu, D. R. Yu, J. Yu, J. Yu, L. Yuan, A. Yurkewicz, B. Zabinski, R. Zaidan, A. M. Zaitsev, L. Zanello, D. Zanzi, A. Zaytsev, C. Zeitnitz, M. Zeman, A. Zemla, O. Zenin, T. Ženiš, Z. Zinonos, D. Zerwas, G. Zevi della Porta, D. Zhang, H. Zhang, J. Zhang, X. Zhang, Z. Zhang, L. Zhao, Z. Zhao, A. Zhemchugov, J. Zhong, B. Zhou, N. Zhou, Y. Zhou, C. G. Zhu, H. Zhu, J. Zhu, Y. Zhu, X. Zhuang, V. Zhuravlov, A. Zibell, D. Zieminska, N. I. Zimin, R. Zimmermann, S. Zimmermann, S. Zimmermann, M. Ziolkowski, R. Zitoun, L. Živković, V. V. Zmouchko, G. Zobernig, A. Zoccoli, M. zur Nedden, V. Zutshi, L. Zwalinski

**Affiliations:** 1 CERN, 1211 Geneva 23, Switzerland; 2 School of Chemistry and Physics, University of Adelaide, Adelaide, Australia; 3 Physics Department, SUNY Albany, Albany, NY United States of America; 4 Department of Physics, University of Alberta, Edmonton, AB Canada; 5 Department of Physics, Ankara University, Ankara, Turkey; 6 Department of Physics, Dumlupinar University, Kutahya, Turkey; 7 Department of Physics, Gazi University, Ankara, Turkey; 8 Division of Physics, TOBB University of Economics and Technology, Ankara, Turkey; 9 Turkish Atomic Energy Authority, Ankara, Turkey; 10 LAPP, CNRS/IN2P3 and Université de Savoie, Annecy-le-Vieux, France; 11 High Energy Physics Division, Argonne National Laboratory, Argonne, IL United States of America; 12 Department of Physics, University of Arizona, Tucson, AZ United States of America; 13 Department of Physics, The University of Texas at Arlington, Arlington, TX United States of America; 14 Physics Department, University of Athens, Athens, Greece; 15 Physics Department, National Technical University of Athens, Zografou, Greece; 16 Institute of Physics, Azerbaijan Academy of Sciences, Baku, Azerbaijan; 17 Institut de Física d’Altes Energies and Departament de Física de la Universitat Autònoma de Barcelona and ICREA, Barcelona, Spain; 18 Institute of Physics, University of Belgrade, Belgrade, Serbia; 19 Vinca Institute of Nuclear Sciences, University of Belgrade, Belgrade, Serbia; 20 Department for Physics and Technology, University of Bergen, Bergen, Norway; 21 Physics Division, Lawrence Berkeley National Laboratory and University of California, Berkeley, CA United States of America; 22 Department of Physics, Humboldt University, Berlin, Germany; 23 Albert Einstein Center for Fundamental Physics and Laboratory for High Energy Physics, University of Bern, Bern, Switzerland; 24 School of Physics and Astronomy, University of Birmingham, Birmingham, United Kingdom; 25 Department of Physics, Bogazici University, Istanbul, Turkey; 26 Division of Physics, Dogus University, Istanbul, Turkey; 27 Department of Physics Engineering, Gaziantep University, Gaziantep, Turkey; 28 Department of Physics, Istanbul Technical University, Istanbul, Turkey; 29 INFN Sezione di Bologna, Bologna, Italy; 30 Dipartimento di Fisica, Università di Bologna, Bologna, Italy; 31 Physikalisches Institut, University of Bonn, Bonn, Germany; 32 Department of Physics, Boston University, Boston, MA United States of America; 33 Department of Physics, Brandeis University, Waltham, MA United States of America; 34 Universidade Federal do Rio De Janeiro COPPE/EE/IF, Rio de Janeiro, Brazil; 35 Federal University of Juiz de Fora (UFJF), Juiz de Fora, Brazil; 36 Federal University of Sao Joao del Rei (UFSJ), Sao Joao del Rei, Brazil; 37 Instituto de Fisica, Universidade de Sao Paulo, Sao Paulo, Brazil; 38 Physics Department, Brookhaven National Laboratory, Upton, NY United States of America; 39 National Institute of Physics and Nuclear Engineering, Bucharest, Romania; 40 University Politehnica Bucharest, Bucharest, Romania; 41 West University in Timisoara, Timisoara, Romania; 42 Departamento de Física, Universidad de Buenos Aires, Buenos Aires, Argentina; 43 Cavendish Laboratory, University of Cambridge, Cambridge, United Kingdom; 44 Department of Physics, Carleton University, Ottawa, ON Canada; 45 CERN, Geneva, Switzerland; 46 Enrico Fermi Institute, University of Chicago, Chicago, IL United States of America; 47 Departamento de Física, Pontificia Universidad Católica de Chile, Santiago, Chile; 48 Departamento de Física, Universidad Técnica Federico Santa María, Valparaíso, Chile; 49 Institute of High Energy Physics, Chinese Academy of Sciences, Beijing, China; 50 Department of Modern Physics, University of Science and Technology of China, Anhui, China; 51 Department of Physics, Nanjing University, Jiangsu, China; 52 School of Physics, Shandong University, Shandong, China; 53 Physics Department, Shanghai Jiao Tong University, Shanghai, China; 54 Laboratoire de Physique Corpusculaire, Clermont Université and Université Blaise Pascal and CNRS/IN2P3, Clermont-Ferrand, France; 55 Nevis Laboratory, Columbia University, Irvington, NY United States of America; 56 Niels Bohr Institute, University of Copenhagen, Kobenhavn, Denmark; 57 INFN Gruppo Collegato di Cosenza, Arcavata di Rende, Italy; 58 Dipartimento di Fisica, Università della Calabria, Arcavata di Rende, Italy; 59 Faculty of Physics and Applied Computer Science, AGH University of Science and Technology, Krakow, Poland; 60 The Henryk Niewodniczanski Institute of Nuclear Physics, Polish Academy of Sciences, Krakow, Poland; 61 Physics Department, Southern Methodist University, Dallas, TX United States of America; 62 Physics Department, University of Texas at Dallas, Richardson, TX United States of America; 63 DESY, Hamburg and Zeuthen, Germany; 64 Institut für Experimentelle Physik IV, Technische Universität Dortmund, Dortmund, Germany; 65 Institut für Kern- und Teilchenphysik, Technical University Dresden, Dresden, Germany; 66 Department of Physics, Duke University, Durham, NC United States of America; 67 SUPA - School of Physics and Astronomy, University of Edinburgh, Edinburgh, United Kingdom; 68 INFN Laboratori Nazionali di Frascati, Frascati, Italy; 69 Fakultät für Mathematik und Physik, Albert-Ludwigs-Universität, Freiburg, Germany; 70 Section de Physique, Université de Genève, Geneva, Switzerland; 71 INFN Sezione di Genova, Genova, Italy; 72 Dipartimento di Fisica, Università di Genova, Genova, Italy; 73 E. Andronikashvili Institute of Physics, Iv. Javakhishvili Tbilisi State University, Tbilisi, Georgia; 74 High Energy Physics Institute, Tbilisi State University, Tbilisi, Georgia; 75 II Physikalisches Institut, Justus-Liebig-Universität Giessen, Giessen, Germany; 76 SUPA - School of Physics and Astronomy, University of Glasgow, Glasgow, United Kingdom; 77 II Physikalisches Institut, Georg-August-Universität, Göttingen, Germany; 78 Laboratoire de Physique Subatomique et de Cosmologie, Université Joseph Fourier and CNRS/IN2P3 and Institut National Polytechnique de Grenoble, Grenoble, France; 79 Department of Physics, Hampton University, Hampton, VA United States of America; 80 Laboratory for Particle Physics and Cosmology, Harvard University, Cambridge, MA United States of America; 81 Kirchhoff-Institut für Physik, Ruprecht-Karls-Universität Heidelberg, Heidelberg, Germany; 82 Physikalisches Institut, Ruprecht-Karls-Universität Heidelberg, Heidelberg, Germany; 83 ZITI Institut für technische Informatik, Ruprecht-Karls-Universität Heidelberg, Mannheim, Germany; 84 Faculty of Applied Information Science, Hiroshima Institute of Technology, Hiroshima, Japan; 85 Department of Physics, Indiana University, Bloomington, IN United States of America; 86 Institut für Astro- und Teilchenphysik, Leopold-Franzens-Universität, Innsbruck, Austria; 87 University of Iowa, Iowa City, IA United States of America; 88 Department of Physics and Astronomy, Iowa State University, Ames, IA United States of America; 89 Joint Institute for Nuclear Research, JINR Dubna, Dubna, Russia; 90 KEK, High Energy Accelerator Research Organization, Tsukuba, Japan; 91 Graduate School of Science, Kobe University, Kobe, Japan; 92 Faculty of Science, Kyoto University, Kyoto, Japan; 93 Kyoto University of Education, Kyoto, Japan; 94 Department of Physics, Kyushu University, Fukuoka, Japan; 95 Instituto de Física La Plata, Universidad Nacional de La Plata and CONICET, La Plata, Argentina; 96 Physics Department, Lancaster University, Lancaster, United Kingdom; 97 INFN Sezione di Lecce, Lecce, Italy; 98 Dipartimento di Matematica e Fisica, Università del Salento, Lecce, Italy; 99 Oliver Lodge Laboratory, University of Liverpool, Liverpool, United Kingdom; 100 Department of Physics, Jožef Stefan Institute and University of Ljubljana, Ljubljana, Slovenia; 101 School of Physics and Astronomy, Queen Mary University of London, London, United Kingdom; 102 Department of Physics, Royal Holloway University of London, Surrey, United Kingdom; 103 Department of Physics and Astronomy, University College London, London, United Kingdom; 104 Laboratoire de Physique Nucléaire et de Hautes Energies, UPMC and Université Paris-Diderot and CNRS/IN2P3, Paris, France; 105 Fysiska institutionen, Lunds universitet, Lund, Sweden; 106 Departamento de Fisica Teorica C-15, Universidad Autonoma de Madrid, Madrid, Spain; 107 Institut für Physik, Universität Mainz, Mainz, Germany; 108 School of Physics and Astronomy, University of Manchester, Manchester, United Kingdom; 109 CPPM, Aix-Marseille Université and CNRS/IN2P3, Marseille, France; 110 Department of Physics, University of Massachusetts, Amherst, MA United States of America; 111 Department of Physics, McGill University, Montreal, QC Canada; 112 School of Physics, University of Melbourne, Victoria, Australia; 113 Department of Physics, The University of Michigan, Ann Arbor, MI United States of America; 114 Department of Physics and Astronomy, Michigan State University, East Lansing, MI United States of America; 115 INFN Sezione di Milano, Milano, Italy; 116 Dipartimento di Fisica, Università di Milano, Milano, Italy; 117 B.I. Stepanov Institute of Physics, National Academy of Sciences of Belarus, Minsk, Republic of Belarus; 118 National Scientific and Educational Centre for Particle and High Energy Physics, Minsk, Republic of Belarus; 119 Department of Physics, Massachusetts Institute of Technology, Cambridge, MA United States of America; 120 Group of Particle Physics, University of Montreal, Montreal, QC Canada; 121 P.N. Lebedev Institute of Physics, Academy of Sciences, Moscow, Russia; 122 Institute for Theoretical and Experimental Physics (ITEP), Moscow, Russia; 123 Moscow Engineering and Physics Institute (MEPhI), Moscow, Russia; 124 D.V.Skobeltsyn Institute of Nuclear Physics, M.V.Lomonosov Moscow State University, Moscow, Russia; 125 Fakultät für Physik, Ludwig-Maximilians-Universität München, München, Germany; 126 Max-Planck-Institut für Physik (Werner-Heisenberg-Institut), München, Germany; 127 Nagasaki Institute of Applied Science, Nagasaki, Japan; 128 Graduate School of Science and Kobayashi–Maskawa Institute, Nagoya University, Nagoya, Japan; 129 INFN Sezione di Napoli, Napoli, Italy; 130 Dipartimento di Scienze Fisiche, Università di Napoli, Napoli, Italy; 131 Department of Physics and Astronomy, University of New Mexico, Albuquerque, NM United States of America; 132 Institute for Mathematics, Astrophysics and Particle Physics, Radboud University Nijmegen/Nikhef, Nijmegen, Netherlands; 133 Nikhef National Institute for Subatomic Physics and University of Amsterdam, Amsterdam, Netherlands; 134 Department of Physics, Northern Illinois University, DeKalb, IL United States of America; 135 Budker Institute of Nuclear Physics, SB RAS, Novosibirsk, Russia; 136 Department of Physics, New York University, New York, NY United States of America; 137 Ohio State University, Columbus, OH United States of America; 138 Faculty of Science, Okayama University, Okayama, Japan; 139 Homer L. Dodge Department of Physics and Astronomy, University of Oklahoma, Norman, OK United States of America; 140 Department of Physics, Oklahoma State University, Stillwater, OK United States of America; 141 RCPTM, Palacký University, Olomouc, Czech Republic; 142 Center for High Energy Physics, University of Oregon, Eugene, OR United States of America; 143 LAL, Université Paris-Sud and CNRS/IN2P3, Orsay, France; 144 Graduate School of Science, Osaka University, Osaka, Japan; 145 Department of Physics, University of Oslo, Oslo, Norway; 146 Department of Physics, Oxford University, Oxford, United Kingdom; 147 INFN Sezione di Pavia, Pavia, Italy; 148 Dipartimento di Fisica, Università di Pavia, Pavia, Italy; 149 Department of Physics, University of Pennsylvania, Philadelphia, PA United States of America; 150 Petersburg Nuclear Physics Institute, Gatchina, Russia; 151 INFN Sezione di Pisa, Pisa, Italy; 152 Dipartimento di Fisica E. Fermi, Università di Pisa, Pisa, Italy; 153 Department of Physics and Astronomy, University of Pittsburgh, Pittsburgh, PA United States of America; 154 Laboratorio de Instrumentacao e Fisica Experimental de Particulas - LIP, Lisboa, Portugal; 155 Departamento de Fisica Teorica y del Cosmos and CAFPE, Universidad de Granada, Granada, Spain; 156 Institute of Physics, Academy of Sciences of the Czech Republic, Praha, Czech Republic; 157 Czech Technical University in Prague, Praha, Czech Republic; 158 Faculty of Mathematics and Physics, Charles University in Prague, Praha, Czech Republic; 159 State Research Center Institute for High Energy Physics, Protvino, Russia; 160 Particle Physics Department, Rutherford Appleton Laboratory, Didcot, United Kingdom; 161 Physics Department, University of Regina, Regina, SK Canada; 162 Ritsumeikan University, Kusatsu, Shiga Japan; 163 INFN Sezione di Roma I, Roma, Italy; 164 Dipartimento di Fisica, Università La Sapienza, Roma, Italy; 165 INFN Sezione di Roma Tor Vergata, Roma, Italy; 166 Dipartimento di Fisica, Università di Roma Tor Vergata, Roma, Italy; 167 INFN Sezione di Roma Tre, Roma, Italy; 168 Dipartimento di Fisica, Università Roma Tre, Roma, Italy; 169 Faculté des Sciences Ain Chock, Réseau Universitaire de Physique des Hautes Energies - Université Hassan II, Casablanca, Morocco; 170 Centre National de l’Energie des Sciences Techniques Nucleaires, Rabat, Morocco; 171 Faculté des Sciences Semlalia, Université Cadi Ayyad, LPHEA, Marrakech, Morocco; 172 Faculté des Sciences, Université Mohamed Premier and LPTPM, Oujda, Morocco; 173 Faculté des sciences, Université Mohammed V-Agdal, Rabat, Morocco; 174 DSM/IRFU (Institut de Recherches sur les Lois Fondamentales de l’Univers), CEA Saclay (Commissariat à l’Energie Atomique et aux Energies Alternatives), Gif-sur-Yvette, France; 175 Santa Cruz Institute for Particle Physics, University of California Santa Cruz, Santa Cruz, CA United States of America; 176 Department of Physics, University of Washington, Seattle, WA United States of America; 177 Department of Physics and Astronomy, University of Sheffield, Sheffield, United Kingdom; 178 Department of Physics, Shinshu University, Nagano, Japan; 179 Fachbereich Physik, Universität Siegen, Siegen, Germany; 180 Department of Physics, Simon Fraser University, Burnaby, BC Canada; 181 SLAC National Accelerator Laboratory, Stanford, CA United States of America; 182 Faculty of Mathematics, Physics & Informatics, Comenius University, Bratislava, Slovak Republic; 183 Department of Subnuclear Physics, Institute of Experimental Physics of the Slovak Academy of Sciences, Kosice, Slovak Republic; 184 Department of Physics, University of Johannesburg, Johannesburg, South Africa; 185 School of Physics, University of the Witwatersrand, Johannesburg, South Africa; 186 Department of Physics, Stockholm University, Stockholm, Sweden; 187 The Oskar Klein Centre, Stockholm, Sweden; 188 Physics Department, Royal Institute of Technology, Stockholm, Sweden; 189 Departments of Physics & Astronomy and Chemistry, Stony Brook University, Stony Brook, NY United States of America; 190 Department of Physics and Astronomy, University of Sussex, Brighton, United Kingdom; 191 School of Physics, University of Sydney, Sydney, Australia; 192 Institute of Physics, Academia Sinica, Taipei, Taiwan; 193 Department of Physics, Technion: Israel Institute of Technology, Haifa, Israel; 194 Raymond and Beverly Sackler School of Physics and Astronomy, Tel Aviv University, Tel Aviv, Israel; 195 Department of Physics, Aristotle University of Thessaloniki, Thessaloniki, Greece; 196 International Center for Elementary Particle Physics and Department of Physics, The University of Tokyo, Tokyo, Japan; 197 Graduate School of Science and Technology, Tokyo Metropolitan University, Tokyo, Japan; 198 Department of Physics, Tokyo Institute of Technology, Tokyo, Japan; 199 Department of Physics, University of Toronto, Toronto, ON Canada; 200 TRIUMF, Vancouver, BC Canada; 201 Department of Physics and Astronomy, York University, Toronto, ON Canada; 202 Faculty of Pure and Applied Sciences, University of Tsukuba, Tsukuba, Japan; 203 Department of Physics and Astronomy, Tufts University, Medford, MA United States of America; 204 Centro de Investigaciones, Universidad Antonio Narino, Bogota, Colombia; 205 Department of Physics and Astronomy, University of California Irvine, Irvine, CA United States of America; 206 INFN Gruppo Collegato di Udine, Udine, Italy; 207 ICTP, Trieste, Italy; 208 Dipartimento di Chimica, Fisica e Ambiente, Università di Udine, Udine, Italy; 209 Department of Physics, University of Illinois, Urbana, IL United States of America; 210 Department of Physics and Astronomy, University of Uppsala, Uppsala, Sweden; 211 Instituto de Física Corpuscular (IFIC) and Departamento de Física Atómica, Molecular y Nuclear and Departamento de Ingeniería Electrónica and Instituto de Microelectrónica de Barcelona (IMB-CNM), University of Valencia and CSIC, Valencia, Spain; 212 Department of Physics, University of British Columbia, Vancouver, BC Canada; 213 Department of Physics and Astronomy, University of Victoria, Victoria, BC Canada; 214 Department of Physics, University of Warwick, Coventry, United Kingdom; 215 Waseda University, Tokyo, Japan; 216 Department of Particle Physics, The Weizmann Institute of Science, Rehovot, Israel; 217 Department of Physics, University of Wisconsin, Madison, WI United States of America; 218 Fakultät für Physik und Astronomie, Julius-Maximilians-Universität, Würzburg, Germany; 219 Fachbereich C Physik, Bergische Universität Wuppertal, Wuppertal, Germany; 220 Department of Physics, Yale University, New Haven, CT United States of America; 221 Yerevan Physics Institute, Yerevan, Armenia; 222 Centre de Calcul de l’Institut National de Physique Nucléaire et de Physique des Particules (IN2P3), Villeurbanne, France

## Abstract

A measurement of the top quark pair production cross section in the final state with a hadronically decaying tau lepton and jets is presented. The analysis is based on proton–proton collision data recorded by the ATLAS experiment at the LHC, with a centre-of-mass energy of 7 TeV. The data sample corresponds to an integrated luminosity of 1.67 fb^−1^. The cross section is measured to be $\sigma_{t\bar{t}} = 194 \pm18\ (\mbox{stat}.) \pm46\ (\mbox{syst}.)~\mbox{pb}$ and is in agreement with other measurements and with the Standard Model prediction.

## Introduction

Top quark pairs ($t\bar {t}$) are produced in abundance at the Large Hadron Collider (LHC) due to the high centre-of-mass energy of 7 TeV. The large sample of $t\bar {t}$ events collected with the ATLAS detector makes it possible to study experimentally challenging decay channels and topologies. This letter describes a measurement of the $t\bar {t}$ production cross section. The final state studied here consists of a hadronically decaying tau lepton (*τ*_had_) and jets, corresponding to the $t\bar{t} \rightarrow [b \tau_{\mathrm{had}} \nu_{\tau}][bqq]$ decay, where *b* and *q* are used to denote *b*-quarks and lighter quarks, respectively. Such an event topology with a hadronically decaying tau lepton corresponds to approximately 10 % of all $t\bar {t}$ decays [1].

A $t\bar {t}$ cross-section measurement in the final state with tau leptons makes it possible to probe flavour-dependent effects in top quark decays. It is also relevant to searches for processes beyond the Standard Model, where $t\bar {t}$ events with tau leptons in the final state are a dominant background. This measurement is particularly important for hypothetical charged Higgs boson production [2–5] in top quark decays, where the existence of a charged Higgs boson would lead to an enhancement in the cross section for the considered $t\bar {t}$ final state. The measurement presented here is complementary to the previously published tau + lepton (electron or muon) channel measurement [6]. The most recent cross-section measurements of the tau + jets decay channel have been performed by the CDF and D0 collaborations in proton–antiproton collisions at $\sqrt{s} = 1.96~\mbox{TeV}$ [7, 8]. This is the first measurement reported in this specific channel at the LHC.

In this analysis, events with at least five jets are selected, where two of the jets are identified as having originated from *b*-quarks. After identifying the two jets likely to come from the hadronic decay of one of the top quarks, one of the remaining jets is selected as the *τ*_had_ candidate from the other top quark. The *τ*_had_ contribution is separated from quark- or gluon-initiated jets with a one-dimensional fit to the distribution of the number of tracks associated with the *τ*_had_ candidate. Since the *τ*_had_ decays preferentially to one or three charged particles (and other neutral decay products), this variable provides good separation between hadronically decaying tau leptons and jets, as the latter typically produce a large number of charged particles. The main backgrounds to the $t\bar {t}$ signal are multijet events, $t\bar {t}$ events with a different final state or signal events where the wrong jet is chosen as the *τ*_had_ candidate. A small contribution from single-top and *W*+jets events is also present. The distributions for the backgrounds used in the fit are obtained with data-driven methods.

## The ATLAS detector

The ATLAS detector [9] is a multipurpose particle physics detector with a forward-backward symmetric cylindrical geometry and a near-4*π* coverage in solid angle.^1^ The inner tracking system covers the pseudorapidity range |*η*|<2.5, and consists of a silicon pixel detector, a silicon microstrip detector (SCT), and, for |*η*|<2.0, a transition radiation tracker. The inner detector is surrounded by a thin superconducting solenoid providing a 2 T magnetic field along the beam direction. A high-granularity liquid-argon sampling electromagnetic calorimeter covers the region |*η*|<3.2. An iron/scintillator tile hadronic calorimeter provides coverage in the range |*η*|<1.7. The end-cap and forward regions, spanning 1.5<|*η*|<4.9, are instrumented with liquid-argon calorimeters for both electromagnetic and hadronic measurements. The muon spectrometer surrounds the calorimeters. It consists of three large air-core superconducting toroid systems and separate trigger and high-precision tracking chambers providing accurate muon tracking for |*η*|<2.7.

## Data and simulation samples

The data used in this analysis were collected during the first half of the 2011 data-taking period and correspond to a total integrated luminosity of $\mathcal{L} = 1.67~\mbox{\mbox {fb$^{-1}$}}$. The data sample was selected with a *b*-jet trigger that required at least four jets identified with |*η*|<3.2 and a transverse energy (*E*_T_) above 10 GeV. Two of these jets were required to be identified as *b*-jets using a dedicated high-level-trigger *b*-tagging algorithm [10]. This trigger was enabled for only part of the 2011 data-taking period and is therefore the limiting factor in determining the integrated luminosity of the dataset used.

The selection efficiency for the $t\bar {t} \to \tau_{\mathrm{had}} + \mbox{jets}$ signal is derived from Monte Carlo (MC) simulations. The MC@NLO v4.01 [11] generator, with the parton distribution function (PDF) set CT10 [12], is used for the $t\bar {t}$ signal. The theoretical prediction of the $t\bar {t}$ cross section for proton–proton collisions at a centre-of-mass energy of $\sqrt{s} = 7~\mbox{TeV}$ is $\sigma_{ t\bar {t} } = 167^{+17}_{-18}~\mbox{pb}$ for a top quark mass of 172.5 GeV. It has been calculated at approximate next-to-next-to-leading order (NNLO) in QCD with Hathor 1.2 [13] using the MSTW2008 90 % confidence level NNLO PDF sets [14], incorporating PDF and *α*_*S*_ uncertainties according to the MSTW prescription [15], and cross-checked with the next-to-leading-order + next-to-next-to-leading-log calculation of Cacciari et al. [16] as implemented in Top++ 1.0 [17]. Tau lepton decays are modelled with Tauola [18]. Samples of simulated events are also used to estimate the small contributions from *W*+jets, *Z*+jets, single-top-quark and diboson events, as described in Ref. [19]. The generated events were processed through the full ATLAS detector simulation using Geant4 [20, 21], followed by the trigger and offline reconstruction. The distribution of the number of pile-up events (i.e. collisions in the same, or nearby, bunch crossing as the hard-scattering event) is adjusted to match the scattering multiplicity measured in the data.

## Event selection

Jets are reconstructed from clusters of calorimeter cells [22] using the anti-*k*_*t*_ algorithm [23, 24] with a radius parameter *R*=0.4. The jets are calibrated using transverse momentum- and *η*-dependent corrections obtained from simulation and validated with collision data [25]. Candidate events are required to contain at least five jets with a transverse momentum (*p*_T_) larger than 20 GeV and |*η*|<2.5.

The identification of jets originating from *b*-quarks is performed using algorithms that combine secondary vertex properties and track impact parameters [26]. The algorithm identifies *b*-jets with an average efficiency of 60 % and provides a light-quark jet rejection factor of about 340 in $t\bar {t}$ topologies. The likelihood of misidentifying a *τ*_had_ as a *b*-jet in a $t\bar {t}$ event is approximately 5 %. The two jets with the highest *b*-tag probability are chosen as the event *b*-candidates; events with fewer than two *b*-jets are rejected.

The magnitude of the missing transverse energy ($E_{\mathrm {T}}^{\mathrm {miss}}$) is reconstructed from energy clusters in the calorimeters. The calibration of each cluster depends on the type of physical object associated with the cluster. The transverse momentum of muons in the event is also taken into account. The $E_{\mathrm {T}}^{\mathrm {miss}}$ significance ($S_{E_{\mathrm{T}}^{\mathrm{miss}}}$) is defined as $E_{\mathrm {T}}^{\mathrm {miss}} / (0.5~[\sqrt{\mathrm{GeV}}] \cdot\sqrt{ \sum E_{\mathrm{T}} })$, where ∑*E*_T_ is the scalar sum of the transverse momentum of all objects. Using a $S_{E_{\mathrm{T}}^{\mathrm{miss}}}$ requirement instead of a direct $E_{\mathrm {T}}^{\mathrm {miss}}$ requirement allows the rejection of multijet events where the $E_{\mathrm {T}}^{\mathrm {miss}}$ arises from energy resolution effects, while still retaining high efficiency for signal events with $E_{\mathrm {T}}^{\mathrm {miss}}$ coming from particles which do not interact with the detector [27]. Candidate events are required to have $S_{E_{\mathrm{T}}^{\mathrm{miss}}} > 8$.

Events containing a reconstructed electron or muon [28, 29] with *p*_T_>15 GeV and |*η*|<2.5 are vetoed to reduce the background due to events containing *W* bosons that decay to electrons or muons and to avoid overlap with other $t\bar {t}$ cross-section measurements.

In each event, a single *τ*_had_ candidate is selected from the reconstructed jets using the following procedure. First, the reconstruction of the hadronically decaying top quark is attempted by selecting the three jets (including exactly one of the two *b*-candidates) which, when their four-momenta are added vectorially, give the highest *p*_T_ sum. The remaining jet with the highest *p*_T_, excluding the remaining *b*-candidate, is selected as the *τ*_had_ candidate. Events where the *τ*_had_ candidate *p*_T_ is below 40 GeV are rejected.

The main contributions to the selected *τ*_had_ candidates in the signal region come from the signal (*τ*_had_ from $t\bar {t}$ events), electrons from $t\bar {t}$ events and misidentified jets from $t\bar {t}$, single-top-quark production, *W*+jets and multijet events. The contributions from *Z*/*γ* ^⋆^+jets and diboson processes are negligible.

## Data analysis

The majority of *τ*_had_ decays are characterised by the presence of one or three charged hadrons in the final state, which can be reconstructed as charged particle tracks in the inner detector. The number of tracks (*n*_track_) originating from the interaction point associated with a *τ*_had_ candidate is used to separate the *τ*_had_ contribution from the misidentified jet background.

**Fig. 1:**
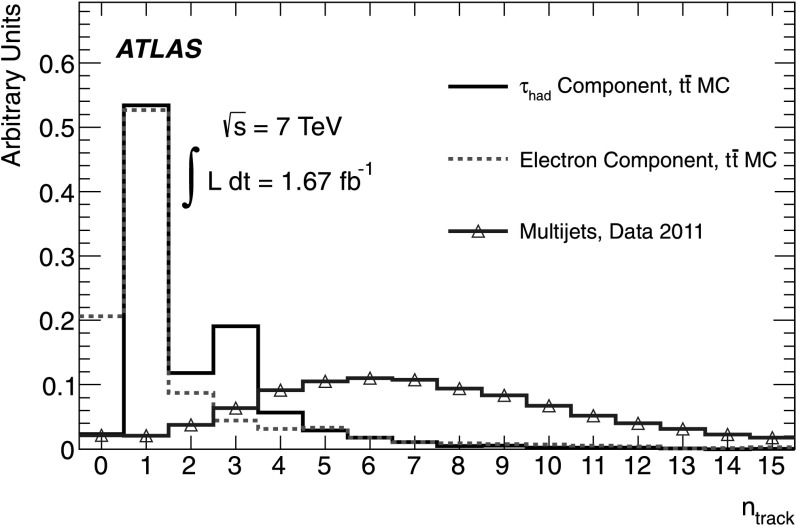
Distribution of *n*_track_ for *τ*_had_ from MC $t\bar {t}$ events (*solid black line*), electrons from MC $t\bar {t}$ events (*dashed red line*), and for jets from multijet events from data (*blue triangles*). The multijet event selection uses a $S_{E_{\mathrm{T}}^{\mathrm{miss}}}$ sideband region as described in Sect. 5. All distributions are normalised to unity (Color figure online)

All selected tracks with *p*_T_>1 GeV located in a core region spanning Δ*R*<0.2 around the jet axis are counted. To increase the discriminating power, tracks in the outer cone 0.2<Δ*R*<0.6 are also counted, using a variable *p*_T_ requirement that is dependent on both the Δ*R* of the outer track and the *p*_T_ of the core tracks. This variable *p*_T_ requirement is designed to reduce the contribution from pile-up and underlying event tracks, and is explained in Ref. [30]. The separation power of the *n*_track_ variable is illustrated in Fig. 1 where a comparison of the *n*_track_ distribution is shown for *τ*_had_, electrons and misidentified jets from multijet events.

To extract the signal from the *n*_track_ distribution, the data sample is fitted with three probability density functions (templates): a *tau/electron* template, a *gluon-jet* template and a *quark-jet* template. The *τ*_had_ component from $t\bar {t}$ events constitutes the signal in the event sample. Real electrons from $t\bar {t}$ events (either prompt or from leptonic tau decays) which failed to be rejected by the electron veto also contribute significantly to the event sample. The electron and *τ*_had_ templates are combined into a single *tau/electron* template to ensure a stable fit, using MC predictions to determine their relative contributions. The *tau/electron* template is obtained from simulated $t\bar {t}$ events. The small expected contributions to the real *tau/electron* component of the fit from single-top-quark and *W*+jets events do not change the shape of the template.

The remaining significant contributions come from misidentified jets, and are separated into two templates. The *gluon-jet* template describes the QCD multijet processes which are dominated by gluon-initiated jets, and the *quark-jet* template describes the remaining processes ($t\bar {t}$, single-top quark and *W*+jets) that are enriched in quark-initiated jets.

The *gluon-jet* template is determined using a sideband region where the $S_{E_{\mathrm{T}}^{\mathrm{miss}}}$ requirement is changed to $3 < S_{E_{\mathrm{T}}^{\mathrm{miss}}} < 4$. This selection greatly enhances the contribution from multijet events, reducing other contributions (e.g. from $t\bar {t}$ events) to less than 1 %. The regions defined by the selection $2 < S_{E_{\mathrm{T}}^{\mathrm{miss}}} < 3$ and $4 < S_{E_{\mathrm{T}}^{\mathrm{miss}}} < 5$ are also used to study any correlations between the $S_{E_{\mathrm{T}}^{\mathrm{miss}}}$ criteria and the *n*_track_ distribution.

The *quark-jet* template is obtained from a $t\bar {t}$ control sample where the *τ*_had_ candidate is replaced by a muon candidate. The reconstructed muon [29] is required to have *p*_T_>20 GeV, |*η*|<2.5 and no jet within a distance Δ*R*=0.4. The requirement on the number of non-*b*-tagged jets is changed from three to two as the jet corresponding to the *τ*_had_ is now replaced by a muon. The other selection requirements are the same as for the signal region. This isolates $t\bar {t}$ events with very high purity; the contribution from backgrounds is estimated from MC predictions to be at the 5 % level, and consists mainly of single-top-quark and *W*+jets events. The two highest-*p*_T_ jets that are not identified as *b*-jet candidates are selected as *τ*_had_ candidates. The template is corrected using MC simulations for differences in the transverse momentum distribution between the signal region and the control sample, and for the expected contribution to the control sample from $t\bar {t}$ dilepton events ($t\bar {t} \rightarrow \mu + \tau_{\mathrm{had}} + X$, $t\bar {t} \rightarrow \mu + e + X$).

## Results

An extended binned-likelihood fit is used to extract the different contributions from the *n*_track_ distribution. To improve the fit stability, a soft constraint is applied to the ratio of *quark-jet* events to *tau/electron* events, which are dominated by the same process ($t\bar {t}$ events). The constraint, based on MC predictions, is a Gaussian with a width of 19 % of its central value. This width was estimated based on studies of the associated systematic uncertainties using the same methodology as described in Sect. 7. The statistical uncertainties on the fit parameters are calculated using the shape of the fit likelihood. The systematic uncertainties on the shapes of the templates are propagated using a pseudo-experiment approach, taking into account the bin-by-bin correlations. This yields a final number of *tau/electron* events of 270 ± 24 (stat.) ± 11 (syst.).

**Fig. 2:**
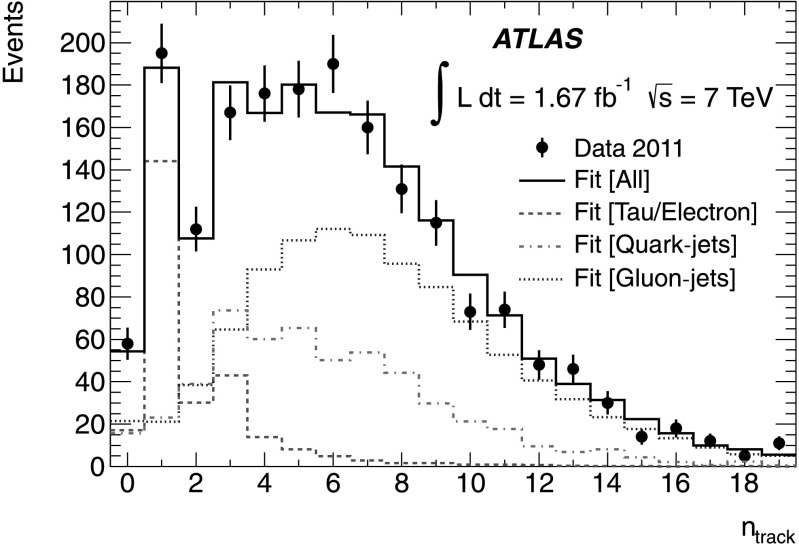
The *n*_track_ distribution for *τ*_had_ candidates after all selection cuts. The black points correspond to data, while the solid black line is the result of the fit. The *red* (*dashed*), *blue* (*dotted*) and *magenta* (*dash-dotted*) *histograms* show the fitted contributions from the *tau/electron* signal, and the *gluon-jet* and *quark-jet* backgrounds, respectively (Color figure online)

The fit results are shown in Fig. 2. A comparison between the fit results, and the expected event yields from the MC predictions is presented in Table 1. The numbers are in good agreement.

**Table 1:** Comparison of the numbers of events from MC expectations and from the results of the fit to the data for the three templates. The uncertainties on the MC expectations include the systematic uncertainties of the selection efficiency described in Sect. 7. No MC predictions are available for the *gluon-jet* contribution

Source	Number of events
*tau/electron*	
$t\bar {t}$ (*τ*_had_)	170 ± 40
$t\bar {t}$ (electrons)	47 ± 11
Single top	12 ± 2
*W*+jets	9 ± 5
Total expected	240 ± 50
Fit result	270 ± 24 (stat.) ± 11 (syst.)
*quark-jet*	
$t\bar {t}$ (jets)	540 ± 160
Single top	24 ± 4
*W*+jets	21 ± 12
Total expected	580 ± 160
Fit result	520 ± 97 (stat.) ± 78 (syst.)
*gluon-jet*	
Fit result	960 ± 77 (stat.) ± 74 (syst.)

To extract the number of signal events, predictions from simulation are used to subtract the backgrounds from *W*+jets and single-top events (9 ± 5 and 12 ± 2, respectively) from the fitted number of *tau/electron* events. The number is then scaled by the expected ratio, *N*_*τ*_/(*N*_*τ*_+*N*_*e*_), of *τ*_had_ and electrons passing the selection in the $t\bar {t}$ sample. This ratio is estimated from MC simulation to be 0.78 ± 0.03 (stat.) ± 0.03 (syst.). This yields a final number of observed signal events of *N*_*τ*_ = 194 ± 18 (stat.) ± 11 (syst.).

The cross section is obtained using $\sigma_{t\bar{t}} = {N_{\tau}}/({\mathcal{L} \cdot \varepsilon})$. The efficiency (*ε*) is estimated from MC simulation to be (6.0 ± 1.4) ×10^−4^. It includes the branching fractions for the various $t\bar {t}$ decays and the acceptance, and assumes Br($t\bar {t}$→*τ*_had_ + jets) to be 0.098 ± 0.002 [1]. The efficiency is corrected for a 13 % difference between MC simulation and data in the trigger and *b*-tagging efficiencies [26]. The method used for obtaining the uncertainty on the cross section is detailed in Sect. 7.

The cross section is measured to be $\sigma_{t\bar{t}}= 194 \pm 18~(\mbox{stat}.) \pm 46~(\mbox{syst}.)~\mbox{pb}$.

## Systematic uncertainties

A summary of all systematic uncertainties on the cross section is given in Table 2.

**Table 2:** Systematic uncertainties on the $t\bar {t}$ cross section

Source of uncertainty	Relative uncertainty
ISR/FSR	15 %
Event generator	11 %
Hadronisation model	6 %
PDFs	2 %
Pile-up	1 %
*b*-jet tagging efficiency	9 %
Jet energy scale	5 %
$E_{\mathrm {T}}^{\mathrm {miss}}$ significance mismodelling	5 %
*b*-jet trigger efficiency	3 %
Jet energy resolution	2 %
Fit systematic uncertainties	4 %
Luminosity	4 %
Total uncertainty	24 %

The uncertainty on the selection efficiency due to the choice of the configuration for the MC simulation is estimated by using alternative MC samples and reweighting procedures. The difference in the efficiency obtained from various configurations is taken as the uncertainty. The uncertainty on the modelling of the ISR/FSR is taken into account by using AcerMC [31] samples with specific tunes aimed at conservatively varying the amount of parton showering [32]. The uncertainty due to the choice of the matrix element event generator is estimated by comparing $t\bar {t}$ samples generated using MC@NLO, Powheg [33–35], and Alpgen [36]. To study the impact of different hadronization models, events generated using Powheg are processed with two different hadronization programs: Herwig and Pythia. The uncertainty of the choice of PDFs is estimated using a number of current PDF sets [37].

Uncertainties on the simulation of the detector response are taken into account using dedicated studies of the reconstructed physics objects (electrons, muons, jets, $E_{\mathrm {T}}^{\mathrm {miss}}$). The uncertainties considered are associated with the jet energy scale, jet energy resolution, *b*-tagging efficiency, trigger efficiency and the $E_{\mathrm {T}}^{\mathrm {miss}}$ calculation [25, 26]. The uncertainty due to mismodelling of the lepton veto is estimated using the uncertainties on the muon and electron reconstruction efficiencies determined from independent data samples, and is found to be negligible.

To obtain the uncertainty on the fit results, variations are applied to the templates to describe various systematic effects. As the *tau/electron* template is taken directly from MC-simulated $t\bar {t}$ events, the systematic uncertainties on this template are taken from estimates of the mismodelling of the simulation. The dominant contributions come from variations in the amount of ISR/FSR in the simulation (1 %), the modelling of the pile-up (1 %), and the statistical uncertainties (1 %). Uncertainties on the track reconstruction efficiency, jet energy scale, and the ratio of *τ*_had_ to electrons are found to be negligible. The *quark-jet* template is obtained from a *μ*+jets control sample of $t\bar {t}$ events in data. The dominant contributions to the uncertainty come from the statistical uncertainties (4 %), the difference in shape between the *μ*+jets template and the expected *quark-jet* distribution, estimated from MC samples (2 %), and the MC-based subtraction of the dilepton contribution (1 %). The uncertainty on the MC-based kinematic correction is found to be negligible. The *gluon-jet* template is derived from a background-dominated sideband region with small values of $S_{E_{\mathrm{T}}^{\mathrm{miss}}}$. The two sources of uncertainties are the dependence of the template on the $S_{E_{\mathrm{T}}^{\mathrm{miss}}}$ criterion of the control region, obtained by varying the $S_{E_{\mathrm{T}}^{\mathrm{miss}}}$ requirement (1 %), and the statistical uncertainty of the control region (1 %). The total systematic uncertainty on the fit is found to be 4 %.

The uncertainty on the luminosity is calculated to be 3.7 % as described in Ref. [38]. The total systematic uncertainty on the cross section is 24 %.

## Conclusions

This letter presents a measurement of the top quark pair production cross section in the final state corresponding to the $t\bar{t} \rightarrow [b \tau_{\mathrm{had}} \nu_{\tau}][bqq]$ decay. The measurement uses a dataset corresponding to an integrated luminosity of 1.67 fb^−1^ of proton–proton collision data at a centre-of-mass energy of 7 TeV recorded by the ATLAS experiment at the LHC. The signal has been extracted by fitting the number of tracks associated with tau lepton candidates using templates derived from simulation for the $t\bar{t}$ signal and from the data for the backgrounds.

The $t\bar {t}$ production cross section is measured to be $\sigma_{t\bar{t}}= 194 \pm 18\ (\mbox{stat}.) \pm 46\ (\mbox{syst}.)~\mbox{pb}$. This result is compatible with the highest precision ATLAS measurements [39, 40], and with the result of 186 ± 13 (stat.) ± 20 (syst.) ± 7 (lum.) pb obtained in the complementary tau + lepton (electron or muon) channel [6]. It is also in good agreement with the theoretical prediction of $167_{-18}^{+17}~\mbox{pb}$.

## Footnotes

1 ATLAS uses a right-handed coordinate system with its origin at the nominal interaction point (IP) in the centre of the detector and the *z*-axis along the beam pipe. The *x*-axis points from the IP to the centre of the LHC ring, and the *y*-axis points upward. Cylindrical coordinates (*r*,*ϕ*) are used in the transverse (*x*, *y*) plane, *ϕ* being the azimuthal angle around the beam pipe. The pseudorapidity is defined in terms of the polar angle *θ* as *η*=−lntan(*θ*/2). The variable Δ*R* is used to evaluate the distance between objects, and is defined as $\Delta R = \sqrt{(\Delta\phi)^{2} + (\Delta\eta)^{2}}$.
